# Fractality of tics as a quantitative assessment tool for Tourette syndrome

**DOI:** 10.1098/rsif.2021.0742

**Published:** 2022-02-23

**Authors:** Payton Beeler, Nicholas O. Jensen, Soyoung Kim, Amy Robichaux-Viehoever, Bradley L. Schlaggar, Deanna J. Greene, Kevin J. Black, Rajan K. Chakrabarty

**Affiliations:** ^1^ Center for Aerosol Science and Engineering, Department of Energy, Environmental and Chemical Engineering, Washington University in St Louis, St Louis, MO 63110, USA; ^2^ Institute for Public Health, Washington University in St Louis, St Louis, MO 63110, USA; ^3^ Computational and Systems Biology Program, Division of Biology and Biomedical Sciences, Washington University in St Louis, St Louis, MO 63110, USA; ^4^ Department of Psychiatry, Washington University School of Medicine, St Louis, MO 63130, USA; ^5^ Department of Neurology, Washington University School of Medicine, St Louis, MO 63130, USA; ^6^ Department of Radiology, Washington University School of Medicine, St Louis, MO 63130, USA; ^7^ Department of Neuroscience, Washington University School of Medicine, St Louis, MO 63130, USA; ^8^ Kennedy Krieger Institute, Baltimore, MD 21205, USA; ^9^ Departments of Neurology and Pediatrics, Johns Hopkins University School of Medicine, Baltimore, MD 21205, USA; ^10^ Department of Cognitive Science, University of California San Diego, La Jolla, CA 92093, USA

**Keywords:** tics, Tourette syndrome, fractal, ‌provisional tic disorder

## Abstract

Tics manifest as brief, purposeless and unintentional movements or noises that, for many individuals, can be suppressed temporarily with effort. Previous work has hypothesized that the chaotic temporal nature of tics could possess an inherent fractality, that is, have neighbour-to-neighbour correlation at all levels of timescale. However, demonstrating this phenomenon has eluded researchers for more than two decades, primarily because of the challenges associated with estimating the scale-invariant, power law exponent—called the fractal dimension *D*_f_—from fractional Brownian noise. Here, we confirm this hypothesis and establish the fractality of tics by examining two tic time series datasets collected 6–12 months apart in children with tics, using random walk models and directional statistics. We find that *D*_f_ is correlated with tic severity as measured by the YGTTS total tic score, and that *D*_f_ is a sensitive parameter in examining the effect of several tic suppression conditions on the tic time series. Our findings pave the way for using the fractal nature of tics as a robust quantitative tool for estimating tic severity and treatment effectiveness, as well as a possible marker for differentiating typical from functional tics.

## 1. Introduction

Tics are brief, purposeless, unintentional behaviours appearing as repeated movements of skeletal or vocal musculature, affecting more than 20% of all children [1,2]. Approximately 0.5% of children have Tourette syndrome (TS), which is diagnosed when both motor and vocal tics occur over a period of a year or longer [3]. Tic disorders, including TS, are moderately heritable, but despite decades of active scientific research, no consensus has been reached on their pathophysiological foundations [4,5]. The COVID-19 pandemic has resulted in increased symptoms in children diagnosed with tic disorders, as well as an influx of patients with sudden onset of severe tics and tic-like attacks, the latter of which are commonly believed to be functional tic-like movements [6,7]. The similarity of tics and functional tic-like movements, and the variability of symptoms over time in tic disorders make diagnosis of functional tic-like movements a historically challenging task. Current best practice requires extensive expertise with tic disorders, thorough elicitation of historical information from the patient and family, and careful observation of the patient.

**Figure 1.**
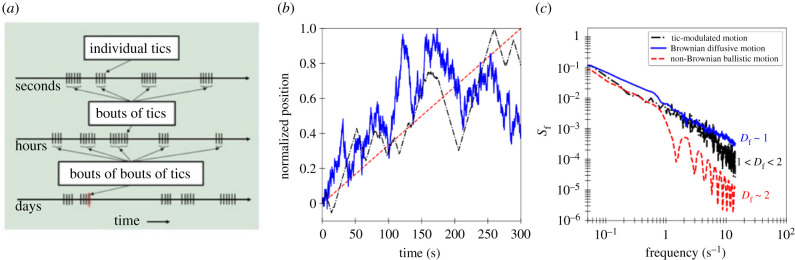
(*a*) Fractal pattern in the occurrence of tics. Tics tend to arise in bouts of several tic occurrences within a few seconds, separated by longer tic-free intervals, and at longer timescales, bouts of tics lasting several seconds similarly recur in grouped episodes over the course of hours. Figure adapted from [9]. (*b*) Trajectory of a tic-modulated random walk (black) is compared with that of Brownian diffusive motion (blue) and non-Brownian ballistic motion (red). The position of each walker at time *t* is normalized by the maximum displacement of the walker, rendering the trajectories in one-dimensional space as a function of time. (*c*) Estimation of *D*_f_ for a walker undergoing diffusive motion (blue), non-Brownian ballistic motion (red) and tic-modulated motion (black).

In 1998, Peterson & Leckman [8] noted that tics tend to arise in clusters (bouts of several tic occurrences within a few seconds, separated by longer tic-free intervals), but also that at longer timescales, bouts of tics lasting several seconds similarly recur in grouped episodes over the course of hours [8,9]. Such recursive behaviour in turn extends to longer time spans (days, weeks and months), maintaining self-similarity (figure 1*a*). Their observation suggests that the chaotic temporal nature of tics could possess an inherent fractality, that is, have neighbour-to-neighbour correlation at all levels of timescale. However, confirming this phenomenon has eluded researchers for more than two decades, primarily because of the challenges associated with estimating the scale-invariant, power law exponent—called the fractal dimension *D*_f_—from fractional Brownian noise.

Here, we uncover the fractal pattern associated with the occurrence of tics from time series datasets collected 6–12 months apart in children. We demonstrate that at timescales ranging from seconds to minutes, the occurrences exhibit neighbour-to-neighbour correlation, and have an associated *D*_f_. The robustness and replicability of *D*_f_ as a quantitative measure lends itself as a simple yet accurate tool for quantifying tic fractality and severity. We are unaware of any previous attempts at estimating *D*_f_ of tics, which would not only provide quantitative insights into the chaotic nature of tic disorders, but also facilitate new ways for assessing tic disorders quantitatively. We also hypothesize that *D*_f_ may be a sensitive parameter in differentiating tics associated with TS from functional tic-like movements.

## 2. Methods

### 2.1. Measuring the time series of tics

The clinical methods appear in detail elsewhere [10]. Briefly, we first recorded the timing of tics in patients during 5 min video sessions corresponding to one of four conditions: free to tic (baseline); verbal request not to tic (verbal); immediate token rewards for 10 s tic-free periods (differential reinforcement of other, ‘DRO’); and tokens given at the same timing as in a previous DRO session regardless of current tic appearance (non-contingent reinforcement, ‘NCR’). Author K.J.B. recorded the timing of tics as they occurred, and rewards were delivered using a custom computer program connected to a token dispenser [11]. In some participants, the NCR condition was omitted, so that most participants had 30–40 min of video at each visit. Video sessions were conducted within the first 6 months after onset of tics (screening visit), when provisional tic disorder (PTD) can be diagnosed, and again at the 12-month anniversary of the first tic (12-month visits), when a chronic tic disorder can be diagnosed. At the 12-month visit, all children still had tics, and most met diagnostic criteria for TS [12]. Data from 78 children were used to generate 535 tic time series during screening visits (164 baseline, 84 NCR, 144 verbal, 143 DRO) and 358 tic time series during 12-month visits (97 baseline, 72 NCR, 94 verbal, 95 DRO). Reproducibility and repeatability of conclusions were confirmed by co-author A.R.V., who independently recorded tic timing from the video recordings blind to visit or video condition [13]. Analysis was then performed on both sets of tic time series.

### 2.2. Modelling tic-modulated motion

Next, we used a random walk model in which the velocity of walkers is reversed with each tic exhibited by the patient, generating what is hereafter referred to as a ‘tic-modulated random walk'. The random walkers are first placed at position *x* = 0, and given an initial velocity of one position unit (arbitrary) per second. Time is then incremented by 0.1 s, and the walker will either reverse velocity or continue with the same velocity as the previous time step, according to a certain probability function (*p*_turn_(*t*)).

Walkers undergoing tic-modulated motion have *p*_turn_(*t*), which is determined by the tic time series. If a tic is not detected at time *t*, then *p*_turn_(*t*) is equal to zero, and the walker continues moving with the same velocity as the previous time step. If a tic is detected at time *t*, then *p*_turn_(*t*) is equal to unity, and the velocity of the walker is reversed. This is summarized by

2.1 $$p_{\mathrm{turn}}(t)\;=\{\;\begin{array}{c} \;\;\;\;\;\;\;0,\;\mathrm{no}\;\mathrm{tic}\;\mathrm{detected}\;\mathrm{at}\;t\\ \;\;\;1,\;\mathrm{tic}\;\mathrm{detected}\;\mathrm{at}\;t \end{array}.$$

Figure 1*b* shows the trajectory of a tic-modulated random walker with data obtained from one child with TS (black line). The trajectory of the tic-modulated random walker is compared with two cases that represent the two extremes of a tic time series. The first case is the most chaotic case for a tic time series, Brownian diffusive motion. Under this regime, *p*_turn_(*t*) is a constant value of 0.5. A tic-modulated time series that closely resembles Brownian diffusive motion indicates that the patient is equally likely to tic or not tic at any given time. The second case is the most ordered case for a tic time series, non-Brownian ballistic motion. Under this regime, *p*_turn_(*t*) is a constant value of zero (i.e. the patient has a 0% chance of exhibiting a tic at any given time). When compared to the trajectory of a walker undergoing Brownian diffusive motion, some excursive episodes of the walker (i.e. longer periods without a tic) can be observed in the tic-modulated trajectories. Qualitatively speaking, the mobility of the tic-driven walker is stronger than that of Brownian diffusive motion but weaker than that of non-Brownian ballistic motion.

### 2.3. Estimation of *D*_f_ using the spectral density function

The fractal dimension of the tic time series was determined by analysing the squared Fourier transform of the density autocorrelation function of the random walkers (*S*_f_) [14], which is given by:

2.2 $$\begin{array}{c} S_{f}={\left|\frac{1}{N}\overset{N}{\underset{\;j\;=1}{\sum }}\exp\;[i(\;f\cdot \overset{⇀}{r_{j}})]\right|}^{2}\;\; \end{array}$$

where${\vec{r}}_{j}$ is the position of the walker at the *j*th time step, *N* is the total number of time steps (3000) and *f* is the frequency [14,15]. Equation (2.2) has been used extensively in engineering to estimate the fractal dimension of objects from their angular scattering pattern. Equation (2.2) has been slightly modified for the purpose of this work; full derivation can be found in [15]. For each random walk trajectory, *S*_f_ was found by rotating the trajectory of the walker by an angle (*θ*), then solving equation (2.2) for a given frequency. The result of equation (2.2) is then averaged over 180 values of *θ* (evenly distributed between 0 and 2π), to give *S*_f_ as a function of frequency. The slope of *S*_f_ versus *f* in log–log space then gives *D*_f_ of the tic time series [14]. It should be noted that this work uses a stochastic model to investigate the dynamics of tic time series at relatively short timescales. Therefore, in the context of this work, *D*_f_ is a local property that is independent of the Hurst coefficient [16]. Figure 1*c* shows examples of *S*_f_ for a tic-modulated random walker, as well as walkers undergoing Brownian diffusion and non-Brownian ballistic motion. Non-Brownian ballistic motion and Brownian diffusion can be, respectively, quantified by *D*_f_ ≈ 2 and *D*_f_ ≈ 1. Brownian diffusive motion and non-Brownian ballistic motion define an expected range for *D*_f_ that correlates with tic severity, and experimentally measured tic time series can be interpreted within this domain.

## 3. Results

**Figure 2.**
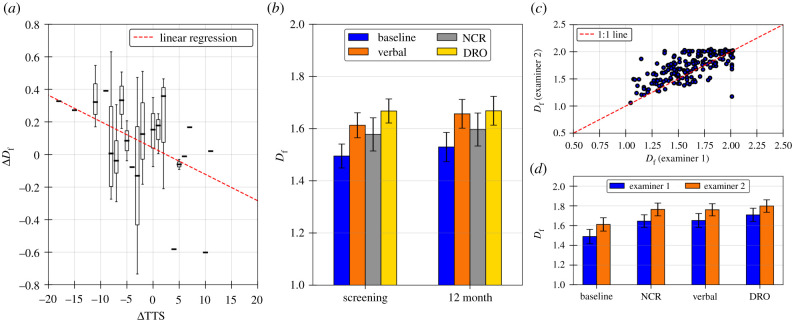
(*a*) Change in fractal dimension between screening and 12-month visit (Δ*D*_f_) as a function of change in YGTSS TTS (ΔTTS). Here, Δ*D*_f_ is calculated using the tic time series under baseline condition. The red dashed line shows linear regression of Δ*D*_f_ versus ΔTTS, *r* = −0.333, *N* = 41. (*b*) Comparison of average *D*_f_ for the patients under various suppression conditions during screening visits shows that DRO led to the most effective tic suppression (largest *D*_f_). Additionally, the fractal dimension of all suppression conditions increased at 12 months (when most patients met diagnostic criteria for TS). Error bars show 95% confidence intervals. (*c*,*d*) Rater-blind reproducibility of *D*_f_ as an assessment tool. Comparison of *D*_f_ for tic time series generated by two examiners, with examiner 2 being blind to visit and condition. Good agreement is observed between examiners, verifying inter-rater reliability of results. Error bars show 95% confidence intervals.

Figure 2*a* shows that for individual patients, the change in *D*_f_ of the tic time series is correlated with change in tic severity, as measured by the Yale Global Tic Severity Scale total tic score (TTS) between screening and 12-month visits (*r* = −0.33, *p* = 0.03). Patients with decreased TTS between screening and 12-month visits (greater improvement in tics) also had increased *D*_f_ of the tic time series. Given these results, *D*_f_ of the tic time series can be used to measure tic severity, with more severe tics having *D*_f_ ≈ 1, and less severe tics having *D*_f_ ≈ 2. Figure 2*b* demonstrates the overall effectiveness of various tic suppression methods using the average *D*_f_ of the tic-modulated walkers. We find that there are statistically significant changes to *D*_f_ depending on suppression condition (*p* = 0.004, using repeated measures ANOVA) [17]. Figure 2*b* shows that during screening visits, DRO and verbal conditions were the most effective suppression techniques, marked by the largest increase in *D*_f_, while NCR's effects on *D*_f_ were not as significant. These findings are consistent with previous results, which show that DRO is an effective suppression technique [10]. Similarly, 12 months after the onset of tics, DRO and verbal suppression conditions led to increases in *D*_f_, and the effect of NCR was not as significant. The reproducibility of our results is demonstrated in figure 2*c,d*. Figure 2*c,d* shows that *D*_f_ of time series generated by examiner 2 was higher on average than *D*_f_ of time series generated by examiner 1. However, *D*_f_ from the two independent raters was highly correlated (*r* = 0.71, *N* = 194 sessions from screening visits), indicating that the fractal dimension can be used to draw objective conclusions regarding tic severity**.**

## 4. Discussion

In the present study, we validate the hypothesis of fractal timing of tics in TS first reported over 20 years ago [8] and extend that observation for the first time to tics shortly after they appear (PTD). We first show that *D*_f_ of the tic time series is correlated with a standard clinical measure of tic severity (TTS), suggesting that *D*_f_ of the tic time series can be used as an objective measure of tic severity. Additionally, we measure for the first time the effect of tic suppression on the temporal dynamics of tic occurrence by quantification of *D*_f_. Using this analytical framework, we further demonstrate that various tic suppression techniques have an effect on this relationship, with increased effectiveness reflected by increased *D*_f_. Overall, DRO and verbal instruction were effective tic suppression conditions during screening and 12-month visits, with DRO being the most effective. In addition, all conditions showed increased *D*_f_ at 12-month visits compared to screening visits, which may be attributed to the passage of time, with continuing cognitive development and additional practice with environmental tic suppression resulting in improved tic inhibition in the social environment. Finally, we demonstrate the robustness of this parameter via the congruence with our results by a blinded rater. Additionally, we also use a stochastic random walk model to observe *D*_f_, which does not require complex mathematical treatment in order to obtain *D*_f_. Future work should be directed to extend the scaling analysis to longer time spans, since qualitatively the temporal dynamics of tics have been observed to maintain self-similarity over months and even years. This method for analysing the timing of tic occurrence shows promise for documenting and understanding tic suppression-based behaviour therapies for TS [18]. We also speculate that tic timing patterns in patients with functional tics may be different than those seen in TS, potentially providing an objective tool to help with diagnosis [6]. Future work will include examination of the behaviour of tics outside of a laboratory setting, and the effects of environmental stressors on tic suppression.

## Data accessibility

All relevant datasets used for this study, including time series of tics and calculated fractal dimensions, can be found in the electronic supplementary material, datasets S1 and S2. The data are provided in the electronic supplementary material [19]. Code is available at https://doi.org/10.5281/zenodo.5949339.
